# Predictive Refined Computational Modeling of ACL Tear Injury Patterns

**DOI:** 10.3390/bioengineering11050413

**Published:** 2024-04-23

**Authors:** Mirit Sharabi, Raz Agron, Amir Dolev, Rami Haj-Ali, Mustafa Yassin

**Affiliations:** 1 Department of Mechanical Engineering and Mechatronics, Ariel University, Ariel 407000, Israel; agronraz@gmail.com; 2 School of Mechanical Engineering, Tel Aviv University, Tel Aviv 69978, Israel; rami98@tau.ac.il; 3 Department of Orthopedics, HaSharon Hospital, Rabin Medical Center, Petach Tikva 49372, Israel; amirdolev1@gmail.com (A.D.); mustafay@clalit.org.il (M.Y.)

**Keywords:** anterior cruciate ligament, finite element, partial tears, Lachman test, anterior tibial translation, composite material

## Abstract

Anterior cruciate ligament (ACL) ruptures are prevalent knee injuries, with approximately 200,000 ruptures annually, and treatment costs exceed USD two billion in the United States alone. Typically, the initial detection of ACL tears and anterior tibial laxity (ATL) involves manual assessments like the Lachman test, which examines anterior knee laxity. Partial ACL tears can go unnoticed if they minimally affect knee laxity; however, they will progress to a complete ACL tear requiring surgical treatment. In this study, a computational finite element model (FEM) of the knee joint was generated to investigate the effect of partial ACL tears under the Lachman test (GNRB^®^ testing system) boundary conditions. The ACL was modeled as a hyperelastic composite structure with a refined representation of collagen bundles. Five different tear types (I–V), classified by location and size, were modeled to predict the relationship between tear size, location, and anterior tibial translation (ATT). The results demonstrated different levels of ATT that could not be manually detected. Type I tears demonstrated an almost linear increase in ATT, with the growth in tear size ranging from 3.7 mm to 4.2 mm, from 25% to 85%, respectively. Type II partial tears showed a less linear incline in ATT (3.85, 4.1, and 4.75 mm for 25%, 55%, and 85% partial tears, respectively). Types III, IV, and V maintained a nonlinear trend, with ATTs of 3.85 mm, 4.2 mm, and 4.95 mm for Type III, 3.85 mm, 4.25 mm, and 5.1 mm for Type IV, and 3.6 mm, 4.25 mm, and 5.3 mm for Type V, for 25%, 55%, and 85% partial tears, respectively. Therefore, for small tears (25%), knee stability was most affected when the tears were located around the center of the ligament. For moderate tears (55%), the effect on knee stability was the greatest for tears at the proximal half of the ACL. However, severe tears (85%) demonstrated considerable growth in knee instability from the distal to the proximal ends of the tissue, with a substantial increase in knee instability around the insertion sites. The proposed model can enhance the characterization of partial ACL tears, leading to more accurate preliminary diagnoses. It can aid in developing new techniques for repairing partially torn ACLs, potentially preventing more severe injuries.

## 1. Introduction

The anterior cruciate ligament (ACL) is a critical ligament that helps stabilize the knee joint’s anterior tibial translation (ATT). ACL ruptures are prevalent, with around 200,000 occurring annually [1], leading to treatment costs exceeding USD two billion in the United States alone [2]. These injuries often happen during sports activities that involve sudden stops, jumping, or sharp changes in direction, such as basketball, football, soccer, and volleyball [3,4,5,6,7]. Once torn, the ACL cannot regrow or heal naturally. ACL injuries are associated with several risk factors, such as female sex, young age, and sports. Bone morphology, neuromuscular control, genetic profile, and hormonal balance may also contribute to ACL injury risk [7].

Such injuries often lead to secondary degenerative changes, long-term functional impairment, and an increased risk of developing osteoarthritis at an earlier age [8]. The primary function of the ACL is to prevent the anteroposterior displacement of the tibia on the femur, providing over 80% of the anterior restraining force from 30° to 90° of knee flexion. Its secondary role is to offer rotational stability [8]. The ACL consists of two bundles: the anteromedial (AM) bundle, which comprises 49% of the femoral footprint and stabilizes the knee during flexion, and the posterolateral (PL) bundle, which accounts for the remaining 51% and is stretched when the knee is fully extended [9,10].

van der List et al. [11] introduced a tear grading system based on tear location, clinical relevance, and MRI feasibility. They examined tear distribution in patients with acute ACL tears from various sports injuries and found that proximal tears were the most prevalent tear type.

Several maneuvers are used to diagnose suspected ACL injuries during a physical examination. In the anterior drawer test, the examiner moves the tibia forward relative to the femur, with the patient’s knee at 90 degrees of flexion and the feet flat; excessive anterior translocation indicates a positive test. More reliable tests include the Lachman and pivot shift tests, which report sensitivities of 0.87 and 0.49, respectively, and specificities of 0.97 and 0.98. Additionally, magnetic resonance imaging (MRI) is strongly recommended as part of the diagnostic evaluation due to its high reported sensitivity (97%) and specificity (100%) in detecting ACL injuries [7].

The Lachman test, which involves manual anterior tibial displacement at 20° of flexion, allows to diagnose ACL tears and anterior tibial laxity (ATL). It is considered the most accurate and reliable clinical test for diagnosing ACL rupture [12]. For more precise ATL assessment, various devices are used, such as the KT-1000™ arthrometer (MEDmetric^®^, San Diego, CA, USA), the Rolimeter™ (Aircast, Summit, NJ, USA), the radiological Telos™ device Telos GmbH, Wölfersheim, Germany), and the GNRB^®^ arthrometer (Genourob, Laval, France) [13,14,15,16].

The GNRB^®^ offers several advantages over other devices, including better accuracy, sensitivity, specificity, and reproducibility in distinguishing between completely torn and intact ACLs [16]. However, it cannot diagnose partial ACL tears, which are of particular interest due to their potential to prevent future total ruptures and subsequent accelerated osteoarthritis [17].

Robert et al. [18] evaluated knee stability using the GNRB^®^ arthrometer, demonstrating its capability to distinguish between healthy, torn, and partially torn ACLs. They applied a linear ascending anterior tibial force and measured the corresponding changes in anterior tibial displacement. Their findings indicated an increase of more than 7 mm in anterior translation between healthy and torn ACLs at 50 N anterior force and a 3 mm increase between partially torn and torn ACLs at the same applied anterior force.

The finite element (FE) method is widely employed in biomechanics to analyze joints and tissues under complex clinically relevant loading conditions. Models of the knee and ACL can incorporate three-dimensional geometry, nonlinear and inhomogeneous material properties, and complex initial boundary and loading conditions. Trad et al. [19] provide a comprehensive description of the FE method for the knee joint. However, despite the potential to obtain crucial insights and a better understanding of ACL ruptures and future treatments, there has been a lack of quantitative investigation of ACL partial tears.

Here, we developed a 3D nonlinear FE model of the knee joint subjected to the Lachman test protocol boundary conditions. We implemented a composite representation of the ACL in the model to investigate the effect of partial ACL tears. First, we validated the anterior tibial translation (ATT) results with GNRB^®^ clinical data [18] for intact, fully, and partially torn ACLs and, then, we used the model to explore different partial tears. These partial tears were divided by severity (indicating tear size, 25, 55, and 85% tear) and type (indicating location (I–V) from the femur to the tibia [11]). Lastly, the influence of the partial tears on the knee laxity, measured by ATT, was quantified.

## 2. Methods

### 2.1. Model Geometry

An FE model of the knee was created based on the open-source 3D knee model (Version 1.0.0, OpenKnee, SimTK, NIH, Cleveland, OH, USA) [20]. The OpenKnee tibiofemoral joint model was based on anatomical (MRI) and mechanical data collected on a cadaver right knee from a 70-year-old female donor as described in [20]. This model geometry was modified using Abaqus FE software (Abaqus 2020, Simulia, Dassault Systèmes, Paris, France) and Ansa CAE pre-processing software (ANSA v20.0.0, BETA CAE Systems SA, Thessaloniki, Greece).

**Figure 1:**
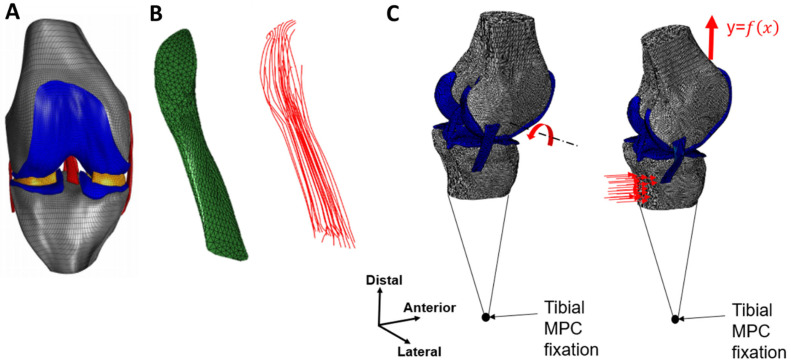
The model and boundary conditions. (**A**) The knee joint model. (**B**) A composite representation of the ACL. (**C**) Boundary conditions applied to the Lachman test simulation.

The model was modified to examine different partial tears in the anterior cruciate ligament (ACL) under the Lachman test boundary conditions. The model geometry consisted of the tibia, tibial cartilage, femur, femoral cartilage, medial collateral ligament (MCL), lateral collateral ligament (LCL), posterior cruciate ligament (PCL), and ACL, as shown in Figure 1A. The menisci were removed to reduce run times and convergence issues caused by the complex interaction between the menisci and cartilages. Their effect on the knee kinematics was simulated using the boundary conditions.

The original model mesh and geometry were adjusted using Ansa software to ensure structural continuity between bone and ligament. This modification unified the bones and ligaments into a single geometry, eliminating singularities in the insertion sites and refining the mesh. Next, surface-to-surface tie interactions linked the cartilages to the femoral and tibial bones. Additionally, node-to-node interactions were implemented at all bone–ligament interfaces.

### 2.2. Composite Representation of the ACL

The initial representation of the ACL in the Lachman model was homogeneous, which was inadequate for modeling partial tears. The ACL isotropic behavior led to inaccuracies, showing near normal stabilization of the joint even when the ligament was almost completely torn. Therefore, a refined hyperelastic composite representation of the ACL was created to mimic its physiological structure, incorporating collagen fibers [21]. This composite ACL was modeled as a biocomposite consisting of collagen fibers reinforcing a proteoglycan matrix with a fiber volume fraction of 60%. This value was evaluated from the higher-end value of the total cross-sectional area occupied by collagen fibrils in the ACL [22]. In the model, the collagen fibers were constructed using truss elements (T3D2H) with a 0.3 mm circular cross-section. Their mechanical behavior was fitted using a 3rd-order Ogden strain energy density function [23] and calibrated to fit healthy ACL experimental results [18]. The proteoglycan matrix was represented with C3D4H tetrahedral solid elements and described using a 1st-order Ogden formulation (Table 1 and Table 2). Hybrid elements were used due to the hyperelastic material properties.

The truss elements were constructed manually by connecting pairs of corresponding nodes of the ACL matrix elements along the path of the ligament, starting from the tibia and ending at the femoral insertion site, creating 27 strings from the 824 truss elements attached between the solid elements’ nodes (Figure 1B). Thus, each two-node truss element shared common nodes with the corresponding solid elements, allowing translation-only load transfer between the matrix and fiber constituents, as previously described [26,28,29,30]. To verify our method for ACL partial tears, we established a sensitivity test to examine the correlation between the geometrical tear size to the ligament cross-section and the percentage of torn fibers required, using nine different tear sizes implemented by deleting elements in the lateral direction of the ligament. Then, we compared the geometric tear and number of torn truss elements by percentile for each tear size and examined the changes in ATT.

### 2.3. Boundary Conditions

Based on the principle of operation of the GNRB knee arthrometer, the boundary conditions were divided into two consecutive steps, as demonstrated in Figure 1C. The first step introduced 20° of flexion to the 3D knee model by rotating the femoral bone around the flexion/extension axis of the knee while fixating the tibial bone to maneuver the joint into the Lachman test position, as performed by the GNRB device. The second step simulated the Lachman test by applying anterior linear pressure to the tibial bone while fixing the tibia translations at a distant point. In the second analysis step, a distal translation for the femur was defined to simulate the menisci effect on the tibiofemoral movement.

### 2.4. Material Properties

The mechanical properties of the model tissues are described in Table 1 and Table 2. The femur and the tibia were modeled as rigid bodies. The ligaments were defined as homogenous, isotropic, and hyperelastic, and their mechanical properties were described using the first-order Ogden material law [23]. The material parameters were computed using curve-fitting data and all ligaments were assumed to be incompressible. The cartilages and bones were described as homogenous and isotropic with linear elastic behavior [24].

The material properties of the composite ACL were based on the literature data [26,27]. The data were calibrated according to the experimental results [18] and implemented in the models.

### 2.5. Lachman Test Model Validation

Three models were generated using a healthy, torn, and partially torn ACL to validate the numerical results. First, a model was created using the healthy ACL to calibrate the properties of the collagen fibers in the ligament by comparing the results to those of the clinical test results obtained by the GNRB arthrometer [18]. Then, the torn and partially torn ACL models were generated accordingly.

Since clinical tear severity or location data were absent, the location was estimated based on the literature [11,31] and an additional model analysis that predicted the critical stress location of the ligament and obtained a partial tear location of approximately 3.7 mm distal to the tibial insertion site. A similar location was also observed in [32]. Therefore, an 85% partial tear was implemented at the approximated location, 3.7 mm from the distal end of the ACL (Type V tear).

### 2.6. Tear Type and Size Model Generation

**Figure 2:**
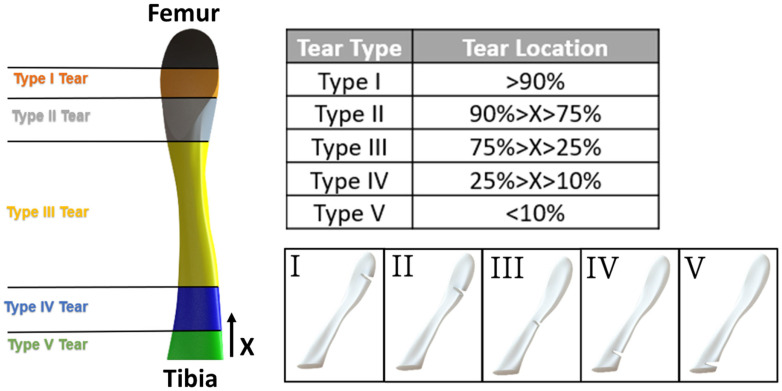
Tear type definitions according to van der List et al. [11].

A location-based classification was proposed for partial tears based on work by van der List et al. (2017) [11]. The partial tears were divided into five types based on their distal distance along the ligament, starting from the tibial insertion site (Figure 2). The ACL length was defined between the center of insertion sites. Tear types were numbered from I to V, where Type I partial tear represented the partial tear closest to the femoral insertion site, and Type V denoted the partial tear closest to the tibial insertion site (Figure 2).

Additional partial tear models (with an acute tear size of 85%) were generated to investigate the effect of tear types (I–V), and the knee stability was evaluated by measuring the ATT for all five tear types. Subsequently, an additional investigation was conducted to explore the effect of the additional tear sizes of 25% and 55%. The effect of tear size on ATT was examined on partial tear sizes of 25%, 55%, and 85% for small, moderate, and acute partial tears, respectively.

## 3. Results

The prediction for the partial tear location was obtained based on maximal Von Mises stress values, similar to the literature [32]. The stress field showed that during the anterior loading of the joint, the healthy ACL developed maximal stresses of 51.2 MPa at the distal part of the ligament near the tibia, compatible with a Type V tear.

**Figure 3:**
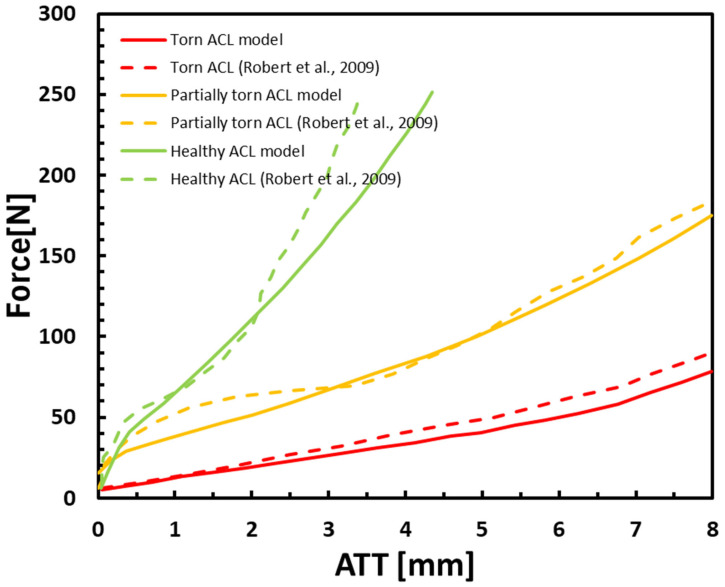
Anterior tibial translation (ATT) vs. anterior force for healthy, torn, and partially torn ACL under the Lachman test. Validated FE model (V type, 85%) vs. clinical data [18]. Solid lines represent the model, and dashed lines represent the clinical data.

The ATT displacements vs. anterior tibial force in an intact partially torn (Type V partial tear 85%), and completely torn ACL are presented in Figure 3. They correlated well with the clinical data [18]. These results demonstrated the ability of the model to differentiate between healthy, torn, and partially torn ACLs by applying a linear ascending anterior tibial force and measuring the corresponding changes to ATT. Our results showed an increase of more than 7 mm to ATT between healthy and torn ACLs at 50 N anterior force and a 3 mm increase between partially torn and torn ACLs at the same applied anterior force.

Our results showed a significant similarity with the clinical data (Figure 3), with an average deviation of 10.7%, 11.2%, and 8.7% for the intact, torn, and partially torn ACL, respectively. Thus, the model can predict the ATT for various degrees of ACL deficiency. The ATT demonstrated a significant difference between all three models. To eliminate the influence of dynamic effects such as bone momentum, the kinetic energy of the model was examined and found to be negligible.

**Figure 4:**
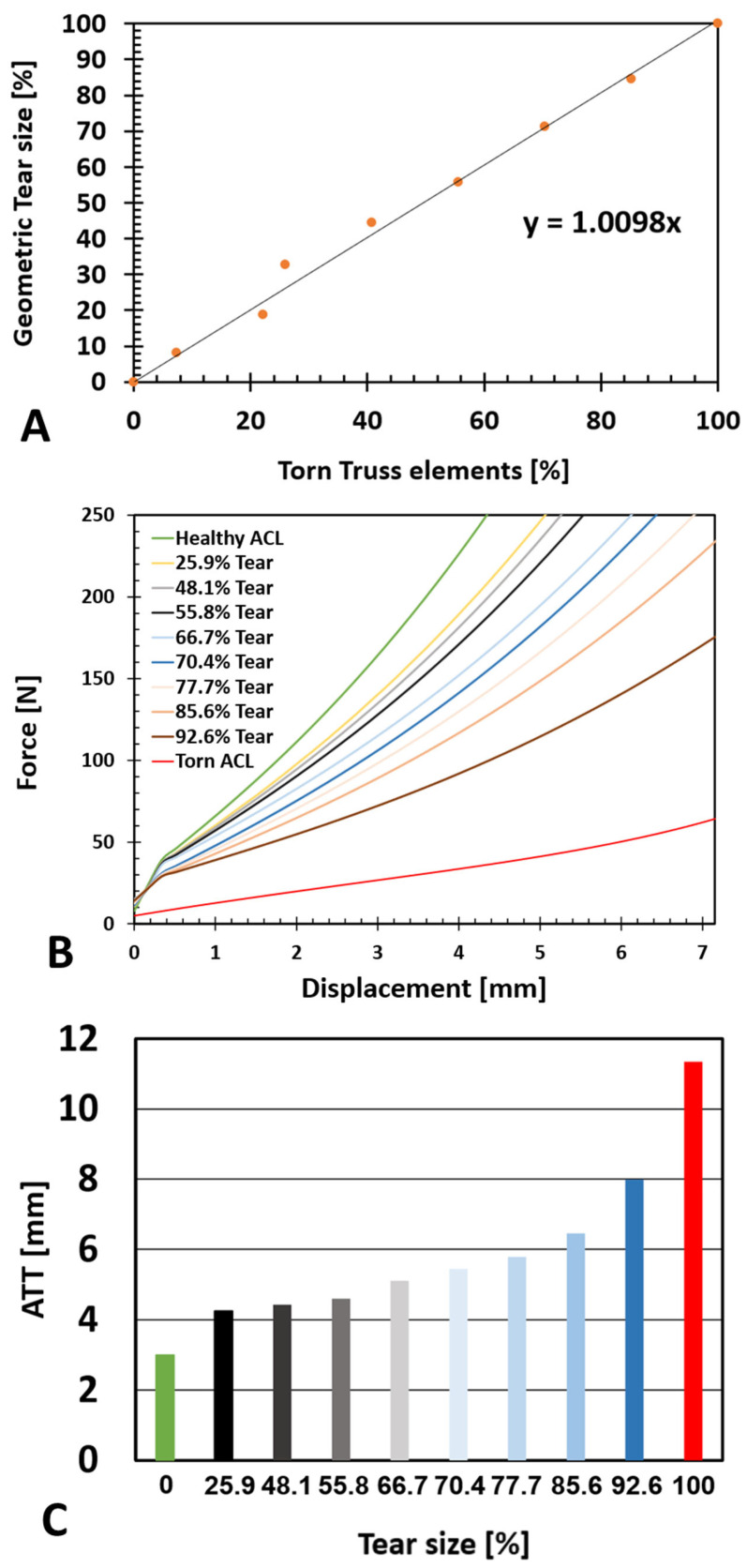
Tear size effect (Type V). (**A**) Correlation of geometric tear size and number of torn truss elements within the composite ACL. (**B**) Anterior tibial displacement in response to a linearly growing anterior force to the tibia for a range of ACL PT sizes, including the limits presented by the healthy and torn ACLs. (**C**) ATT at 200 N for all tested tear sizes exhibiting increased sensitivity for large partial tears.

The correlation between the percentage of the geometric tear size and the number of torn collagen fibers (truss elements) was linear and is presented in Figure 4A. A sensitivity test was conducted to verify the model dependency of the results on nine different tear sizes, from 25.9% to 92.6% for Type V tears (close to the tibia), and to measure their effect on knee stability and ATT (Figure 4).

Figure 4B shows an increase in ATT with an increase in tear size with anterior force up to 250 N. Figure 4C presents the ATT values for all eight tear sizes at a loading of 200 N. An increased effect of tear size growth on knee stability was exhibited when partial tear size grew beyond 75%. Tear sizes below 75% demonstrated a moderate and almost linear trend, with an average increase of 0.05 mm/% tear. However, ATT rose fast in tears above 75%, with an average increase of 0.25 mm/% tear.

**Figure 5:**
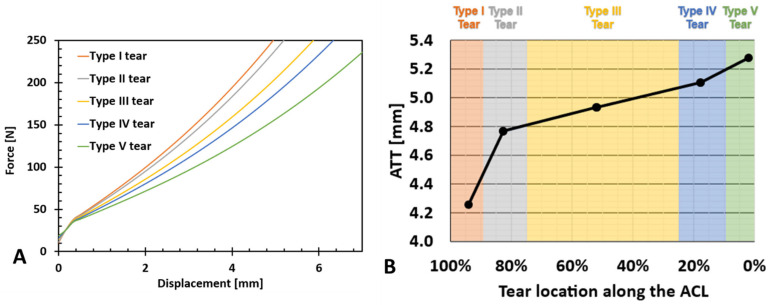
Tear type effect for the 85% tear size. (**A**) Anterior tibial force vs. anterior tibial displacement. The figure reveals a noticeable increase in ATT among tear types and a steady increase with proximity to the tibial insertion site. (**B**) Anterior tibial translation vs. partial tear location along the ACL (LAL). An increase in ATT between tear types at ligament ends is evident.

The effect of partial tear type (or location) on the ATT vs. force for a partial tear of 85% is presented in Figure 5A. When measuring the change at 200 N, the variation in ATT between Type I and Type V partial tears was 1.02 mm, with an average variation of 0.25 mm between adjacent partial tear locations (Figure 5B). However, the effect of tear location (denoted as LAL-location along the ligament) on ATT grew with proximity to the insertion sites. An increase in the slope was observed between tear Types I and II (0.0106 $\frac{mm}{\%LAL}$) and tear Types IV and V (0.0451 $\frac{mm}{\%LAL}$) compared with the slope between tear Types II, III, and IV (0.0052 $\frac{mm}{\%LAL}$) (Figure 5B).

**Figure 6:**
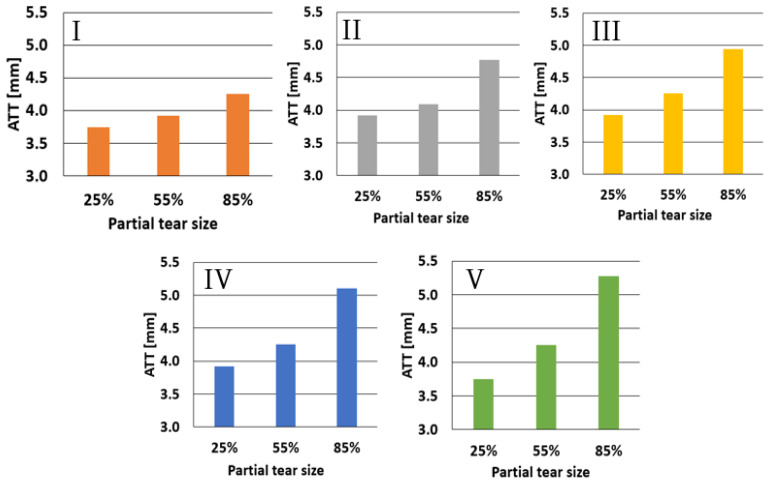
Increased tear size effect on ATT for the different tear types at 200 N anterior force.

The effect of partial tear sizes of 25%, 55%, and 85% for each tear type is shown in Figure 6. Type I tears demonstrated an almost linear increase in ATT, with the growth in tear size ranging from 3.7 mm to 4.2 mm, from 25% to 85%, respectively. Type II partial tears showed a less linear incline in ATT with measured values of 3.85, 4.1, and 4.75 mm for 25%, 55%, and 85% partial tears, respectively. Types III, IV, and V partial tear results maintained the nonlinear trend, with ATTs of 3.85 mm, 4.2 mm, and 4.95 mm for Type III, 3.85 mm, 4.25 mm, and 5.1 mm for Type IV, and 3.6 mm, 4.25 mm, and 5.3 mm for Type V, for 25%, 55%, and 85% partial tears, respectively.

**Figure 7:**
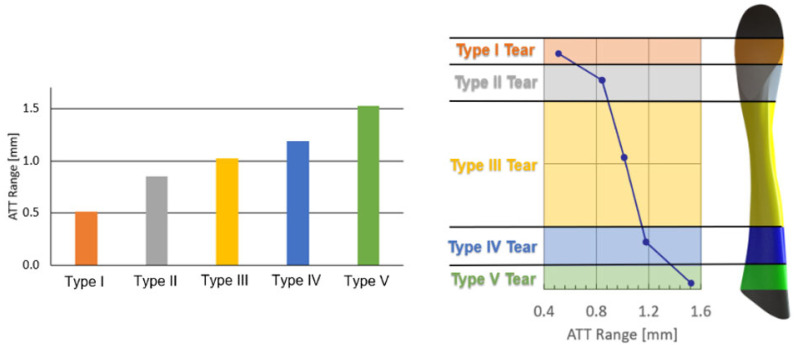
Increase in ATT range with tear growth from 25% to 85%. for the different tear types (**left**). The increase in ATT range with the tear location (**right**).

Figure 7 presents the increase in the ATT range upon tear growth from 25% to 85%. The distal Type I partial tear showed approximately 0.5 mm growth in the anterior tibial laxity due to an increase in tear size, increasing linearly from 3.7 mm for a 25% partial tear to 4.2 mm for an 85% partial tear. The Type II partial tear showed a significant anterior tibial laxity growth of 0.9 mm over the corresponding Type I range. The subsequent ATT growth between Type II and Type III partial tears was 1.1 mm. While moving further toward the tibial insertion site, the ATT grew by an additional 1.25 mm when the tear type changed to Type IV, measuring a moderate linear increase in ATT as the partial tear advanced toward the tibia between tear Types II and IV. Further, the Type V partial tear showed a 1.7 mm rise in the anterior tibial laxity, another significant increase from its adjacent Type IV partial tear. Thus, Type I (near the femur) demonstrated the smallest ATT range increase, while Type V (near the tibia) showed the largest ATT range, exhibiting a trend of increase from the femur to the tibia (I to V tears).

**Figure 8:**
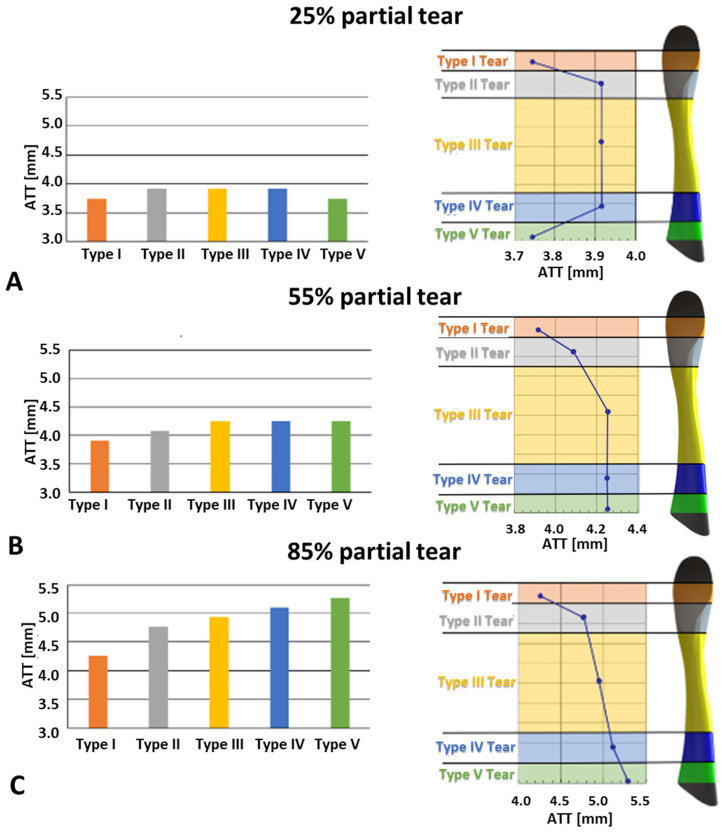
ATT in different tear types at 200 N for partial tears: (**A**) 25%, (**B**) 55%, and (**C**) 85%.

The combined effect of partial tear size with tear location and its influence on the ATT is presented in Figure 8 for partial tear sizes of 25%, 55%, and 85% for the different tear types. The effect of tear location on the ATT varied with its size. For small tears (25%), the effect on the knee stability was mainly around the center of the ligament, with an ATT of 3.92 mm compared with the ATT in the tibial and femoral insertion sites (3.74 mm). For moderate tears (55%), the knee stability was most affected when tears were located at the proximal half of the ACL, with an ATT of 4.23 mm, compared with 3.86 mm ATT at the distal end. However, severe tears (85%) demonstrated considerable growth in knee instability from the distal to the proximal ends of the tissue, with an exceeding increase in knee instability around the insertion sites, yielding an ATT of 4.25 mm for Type I tears, which increased to 4.8 mm in Type II tears. The rise in ATT was approximately linear from Type II to Type IV partial tears, with a Type III partial tear exhibiting an ATT of 4.95 mm and a Type IV ATT of 5.1 mm. The Type V acute partial tear showed an ATT of 5.3 mm (Figure 8).

## 4. Discussion

Knee flexion showed minimal difference in anterior tibial translation (ATT) between intact and torn ACLs, indicating the need for an alternative clinical technique to quantify the influence of ACL partial tears. Therefore, the FE model was created with Lachman test boundary conditions, aiming to establish a measurable parameter for quantifying ACL tears using a clinical test. Methods like GNRB and KT1000 enable the quantification of the ACL tear effect, providing a foundational understanding for evaluating changes in ATT with tear size and location. However, available clinical data on the full behavior of ATT versus load are limited.

The FE model was generated based on typical and common dynamic diagnostic methods for measuring ACL tears, such as the GNRB arthrometer. The healthy ACL model demonstrated similar trends between the FE model and clinical data (Figure 3). They both showed a linear increase in ATT for forces between 0 and 45 N, followed by a local drop in ATT response for forces of 45–50 N. Furthermore, a reduction was observed in the ATT response, starting at a 50 N anterior force.

Additional validation of the ability of our model to predict the effect of the ACL integrity on ATT response to anterior forces was found in the torn and partially torn ACL models (Figure 3). The curves showed acceptable mean deviances from the clinical data (11.2% and 8.7% for the torn and partially torn models, respectively), which were expected, considering the variability in mechanical properties and geometries of human tissues [33,34,35]. Lastly, the torn ACL FE model results did not show a stiff joint behavior under the anterior tibial force of 0–50 N. This validated the behavior governed by the ACL and its effect on the surrounding tissues, affecting the complex movement of the joint when an excessive anterior translation is applied.

A slight offset was observed in the beginning of the curves, for both the clinical data and the FE model (Figure 3). In the model, this offset was a result of applying two-stage boundary conditions (20° flexion and then pressure, as in the clinical GNRB test). Thus, the flexion was also affected by the initial ACL state. However, the offset was even larger in the clinical data. A possible explanation was that the examiner’s experience also affected the results [18]. However, since this offset was seen both in the model and clinical data, it could stem from the ACL intrinsic structure and geometry.

Physiologically, the ACL is separated into two bundles (the anteromedial (AM) and posterolateral (PL) bundles), which twist and untwist with joint motion [10]. The ATT strongly depends on the structure of the ligament, as shown experimentally [36]. Our model represented the ACL as a single part with collagen fiber reinforcements without the physiological separation into its two bundles. However, we demonstrated that our structural representation of the ACL was essential for partial tear investigation and did not depend on the ACL mechanical behavior alone. Furthermore, Hara et al. [21] showed that the deviation for two bundles was insufficient and suggested dividing it into smaller bundles, as in our model. Thus, they separated each anteromedial (AM) and posterolateral (PL) bundle into approximately ten small bundles, similar to our model.

For Type V tears, larger partial tears resulted in a greater anterior tibial translation (ATT) response to anterior forces, thereby increasing knee instability. The relationship between partial tear size and the ATT was non-linear, with tear sizes above 50% showing a more pronounced effect on knee instability. This ~50% tear effect was intriguing, especially considering that the model did not differentiate between the two bundles in the ACL. Anatomically, the ACL comprises the anteromedial (AM) and posterolateral (PL) bundles, which intertwine during knee extension [37]. Therefore, the bundle role appears to be defined by their spatial positions within the ACL. Despite having identical material properties, our findings suggest that the model geometric separation of the bundles was determined by the location of loaded collagen fibers, which was reflected in their influence on knee kinematics.

**Figure 9:**
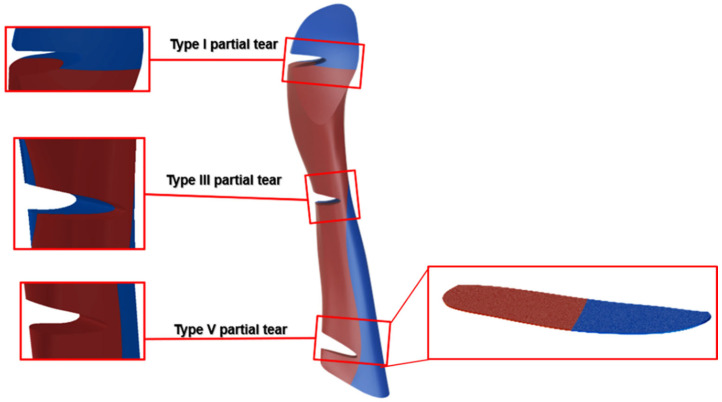
Illustration of the ACL bundle geometry (AL blue and PL red) in different partial tear types, based on the location of ACL partial tears. Type V is the most affected by the partial tear due to the PL location. A zoom-in of the evaluated ACL cross-section for the Type V tear is presented.

Furthermore, the Type V tear was located near the tibial insertion site, starting from the lateral side of the ligament and progressing toward the medial direction. This tear pattern initiated at the location of the posterolateral (PL) bundle and subsequently involved the anteromedial (AM) bundle, as illustrated in the cross-section in Figure 9. Therefore, while the AM bundle was typically associated with knee stabilization during flexion, its stabilizing effect might already have been substantial at a flexion angle of 20°.

Proximal partial tears resulted in greater knee instability compared with distal tears. Additionally, the change in tear location led to more pronounced alterations in ATT measurements near the insertion sites. This more pronounced effect near the insertions suggests their role is not limited only to connective tissues bearing axial tension but also to connective tissues bearing axial tension, as well as to preventing the ACL from untwisting around its axial axis.

The variations in ATT response with changes in tear location at 25% and 55% tear sizes (Figure 6) supported the hypothesis that the ACL bundles played distinct geometrical roles and developed different mechanical properties based on their load-bearing regimes. In small tears, the most significant instability occurred when the tear location was distant from the insertion sites. While we did not find previous data to directly support this premise, the tear location incidence presented by [11] indicated that a false ACL diagnosis as healthy or having a slight strain could occur due to a limited ATT response in small partial tears near the insertion sites.

No significant change in ATT response was observed between partial tear Types II–IV. These results aligned with the current division of the ACL into two bundles, as only a portion of the geometrical PL bundle was torn in tears II, III, and IV. This suggests there was no alteration in load-bearing collagen fibers in these cases. However, in the case of the Type V partial tear, most of the PL bundle was torn, leaving the AM bundle fibers to bear the load, which increased ATT. A schematic representation of PL bundle deficiency due to the partial tear location is provided in Figure 9. For moderate (55% deficient) partial tears, a consistent increase was observed for Types III–V, indicating that the tibial insertion site significantly impacts knee stability in induced tears.

The individual analysis of each tear type suggests that the dependence of knee instability on partial tear size can be defined by its location and growth towards the tibial insertion site (Figure 7). The significant increase in anterior tibial laxity for different tear types raises the possibility of conservative non-operative treatments for minor partial tears in the ACL.

The AM and PL bundles of the ACL are characterized by different injury mechanisms due to their different biomechanical functions. Partial tears of the AM or PL are relatively common (10–35%), with 5–10% of cases being symptomatic. These partial tears can progress to complete tears in 38–50% of patients, often due to failure of blood flow and disruption of the intact bundle. In such cases, ACL augmentation has been shown to be an effective and safe procedure [38].

ACL injuries are common among young athletes, with a recurrence rate of approximately 20% after ACL reconstruction [39]. Therefore, a straightforward method for tracking ACL partial tears could help reduce the need for ACL-related surgical operations and prevent severe injuries. Furthermore, it has been suggested that restoring anterior/posterior and rotational knee laxity could help prevent further instability and subsequent damage to the articular cartilage and meniscus. For example, delayed ACL reconstruction for 5 months doubled the risk of medial meniscal surgery after ACL injury, and if delayed for 1 year, the risk was six times higher [7,40]. Therefore, using our model to predict a specific tear type could enable tear growth tracking to prevent severe injuries to adjacent tissues.

Furthermore, our model suggests that conservative treatment may not be suitable for all partial tear types, as not all types exhibited a significant enough laxity range to be reliably measured. Only tear Types III-V showed a measurable range exceeding 1 mm of anterior tibial translation with increased ACL tear size.

## 5. Limitations

The ACL has an entangled hierarchical structure composed of several structural levels, from the molecular level through the fibril, fiber, and bundle levels, as a nested composite structure [41]. The current analysis could not take into account all structural levels. Furthermore, no specific values represented the collagen fiber volume fraction and the discrete mechanical properties of the fibers and matrix. Thus, these properties were approximated for model simplification using previously published data and our previous models. The material properties were extracted from the literature and fitted to Ogden strain energy density function. These results were based on uniaxial tensile tests of the ligaments and hence could not predict the value of the volumetric part of the Ogden model. Therefore, this value was assumed to be zero, as shown in Table 2. The data inserted into the model were uniaxial stress–strain data that were independent of D. In order to achieve more accurate materials model calibration, additional tissue experiments should be incorporated. However, the data available in the literature are absent. However, for our model, these data were sufficient to acquire the nonlinear stress–strain relationship. Although the use of tetra elements for meshing the ACL was not ideal, it was chosen to allow structural continuity in the insertion sites to avoid singularities and, at the same time, to enable the composite structure of the ACL, consisting of truss and solid elements in specific orientations, that were manually connected. This can be justified since the mesh was refined and our results were comparative.

## 6. Conclusions

Different tear sizes and locations led to varying detectable anterior tibial translation (ATT) levels that could not be manually discerned. Small tears primarily affected knee stability around the ligament center, while moderate tears had the most impact on knee stability when located in the proximal half of the ACL. Severe tears showed substantial growth in knee instability from the distal to the proximal ends of the tissue, with a significant increase in knee instability around the insertion sites.

Therefore, the proposed refined computational model can assist in characterizing partial ACL tears more effectively. It can improve the accuracy of preliminary ACL partial tear diagnoses, enhance partially torn ACL repair techniques, and potentially prevent subsequent severe injuries.

## Figures and Tables

**Table 1:** Model description and material properties.

Part Name		No. of Elements	Element Type	Material Properties	Reference
**Femoral Cartilage**		17,226	C3D8H	Linear elastic E = 5 MPa, υ = 0.46	[24]
**Tibial Cartilage**		8847	C3D8H	Linear elastic E = 5 Mpa, υ = 0.46	[24]
**LCL**		6656	C3D8H	Hyperelastic Ogden (1st order)	[25]
**MCL**		5120	C3D8H	Hyperelastic Ogden (1st order)	[25]
**PCL**		5248	C3D8H	Hyperelastic Ogden (1st order)	[25]
**ACL I**		4096	C3D4H	Hyperelastic Ogden (3rd order)	[25]
**Composite ACL**	**Fibers**	824	T3D2H	Hyperelastic Ogden (3rd order)	Based on [26,27]
	**Matrix**	7412	C3D4H	Hyperelastic Ogden (1st order)	[26]

MCL—medial collateral ligament, LCL—lateral collateral ligament, PCL—posterior cruciate ligament, ACL—anterior cruciate collateral ligament.

**Table 2:** Ogden material parameters.

Part Name		*N*	*i*	$\mu_{\left(i\right)}$ [MPa]	$\alpha_{\left(i\right)}$	$D_{\left(i\right)}$ [MPa^−1^]
**LCL**		**1st order**	1	51.01	19.40	0
**MCL**		**1st order**	1	37.62	24.40	0
**PCL**		**1st order**	1	50.63	16.80	0
**ACL** **Homogeneous (for stress concentration)**		**1st order**	1	84.70	20.48	0
**Composite ACL**	**Collagen Fibers**	**3rd order**	1	−15,890.38	−7.12	0
			2	8450.90	−3.11	0
			3	7455.22	−11.38	0
	**Matrix**	**1st order**	1	0.0473	25	0

MCL—medial collateral ligament, LCL—lateral collateral ligament, PCL—posterior cruciate ligament, ACL—anterior cruciate collateral ligament.

## Data Availability

The original contributions presented in the study are included in the article material, further inquiries can be directed to the corresponding author.
